# The challenges of using radiological ‘tumour response' as an outcome: lessons learned from neo-tango and artemis, two neo-adjuvant chemotherapy breast cancer trials

**DOI:** 10.1186/1745-6215-14-S1-P75

**Published:** 2013-11-29

**Authors:** Louise Hiller, Janet Dunn, Anne-Laure Vallier, Clare Blenkinsop, Louise Grybowicz, Helen Higgins, Helena Earl

**Affiliations:** 1 Warwick Clinical Trials Unit, University of Warwick, Coventry, UK; 2 Cambridge Cancer Trials Centre, Cambridge University Hospitals NHS Foundation Trust, Cambridge, UK; 3 Department of Oncology, University of Cambridge and NIHR Cambridge Biomedical Research Centre, Cambridge, UK

## Objectives

To review the measurement methods of radiological ‘tumour response' in two clinical trials.

## Methods

Neo-tAnGo and ARTemis are national 800-patient breast cancer trials assessing neo-adjuvant chemotherapy regimens, both with a secondary endpoint of radiological tumour response, assessing treatment effect in terms of change in tumour size. Both trials record radiological sizes at baseline, mid-way through and at the end of the chemotherapy regimens.

Neo-tAnGo (randomising Jan'05 to Sept'07) recorded mammograms and/or ultrasounds and/or MRI scans, identifying up to two breast and one axillary lesion as ‘target' lesions. At each of the 3 time-points and for each target lesion, the scan types and largest diameters observed (maximum of two) were reported, along with a clinical judgement of response.

ARTemis (randomising May'09 to Jan'13) recorded ultrasounds only, recording the longest single diameter of each tumour (including axillary tumours) along with total ‘tumour bulk' in each breast. At mid- and end-chemotherapy, a clinical judgement of response per breast is also recorded.

## Results

In neo-tAnGo, the type of radiological scan used and number of recorded dimensions varied, not only across hospitals but also within-hospitals across patients and across assessment times, creating challenges regarding tumour comparisons. In ARTemis, difficulties existed surrounding the clinical response categorisation and interpretation of ‘tumour bulk', and non-adherence to the specified radiological technique raised methodological issues.

## Conclusions

Optimally recording radiological ‘tumour response' and making meaningful inter-patient, inter-tumour or inter-time-point comparisons is challenging. Specifications regarding the authorised radiological scanning techniques and reporting of findings are imperative in a trial protocol.

